# Digital Mental Health Challenges and the Horizon Ahead for Solutions

**DOI:** 10.2196/26811

**Published:** 2021-03-29

**Authors:** Luke Balcombe, Diego De Leo

**Affiliations:** 1 Australian Institute for Suicide Research and Prevention Griffith University Brisbane Australia

**Keywords:** challenges, COVID-19, digital mental health implementation, explainable artificial intelligence, hybrid model of care, human-computer interaction, resilience, technology

## Abstract

The demand outstripping supply of mental health resources during the COVID-19 pandemic presents opportunities for digital technology tools to fill this new gap and, in the process, demonstrate capabilities to increase their effectiveness and efficiency. However, technology-enabled services have faced challenges in being sustainably implemented despite showing promising outcomes in efficacy trials since the early 2000s. The ongoing failure of these implementations has been addressed in reconceptualized models and frameworks, along with various efforts to branch out among disparate developers and clinical researchers to provide them with a key for furthering evaluative research. However, the limitations of traditional research methods in dealing with the complexities of mental health care warrant a diversified approach. The crux of the challenges of digital mental health implementation is the efficacy and evaluation of existing studies. Web-based interventions are increasingly used during the pandemic, allowing for affordable access to psychological therapies. However, a lagging infrastructure and skill base has limited the application of digital solutions in mental health care. Methodologies need to be converged owing to the rapid development of digital technologies that have outpaced the evaluation of rigorous digital mental health interventions and strategies to prevent mental illness. The functions and implications of human-computer interaction require a better understanding to overcome engagement barriers, especially with predictive technologies. Explainable artificial intelligence is being incorporated into digital mental health implementation to obtain positive and responsible outcomes. Investment in digital platforms and associated apps for real-time screening, tracking, and treatment offer the promise of cost-effectiveness in vulnerable populations. Although machine learning has been limited by study conduct and reporting methods, the increasing use of unstructured data has strengthened its potential. Early evidence suggests that the advantages outweigh the disadvantages of incrementing such technology. The limitations of an evidence-based approach require better integration of decision support tools to guide policymakers with digital mental health implementation. There is a complex range of issues with effectiveness, equity, access, and ethics (eg, privacy, confidentiality, fairness, transparency, reproducibility, and accountability), which warrant resolution. Evidence-informed policies, development of eminent digital products and services, and skills to use and maintain these solutions are required. Studies need to focus on developing digital platforms with explainable artificial intelligence–based apps to enhance resilience and guide the treatment decisions of mental health practitioners. Investments in digital mental health should ensure their safety and workability. End users should encourage the use of innovative methods to encourage developers to effectively evaluate their products and services and to render them a worthwhile investment. Technology-enabled services in a hybrid model of care are most likely to be effective (eg, specialists using these services among vulnerable, at-risk populations but not severe cases of mental ill health).

## Introduction

The uncertainties of the COVID-19 pandemic, overlapping issues, and the long-term impact on population mental health make it difficult to predict the issues that would become prevalent. A preliminary longitudinal study reported an increase in the prevalence and incidence of common mental health disorders (not major psychiatric conditions)—mostly related to loneliness—but with a return to prepandemic levels [1]. Chandola et al [1] predicted that unemployment and resulting financial stressors can potentially contribute to an increase in common mental health disorders in the future. Although the initial impact of this pandemic on mental health has been severe, it has been predicted to exacerbate, resulting in an increased number of suicide cases, with the economic impact probably lasting for years [2]. In response to the predicted impact, Rauch et al [3] proposed a method to address mental health needs through a framework of phased interventions and resources with recommendations for leadership and organization within relevant phases. These include self-assessments and brief interventions focused on ensuring that basic needs are met, being creative with socialization, drawing on previously successful positive coping strategies, creating opportunities to communicate and share stories (although avoiding debriefing of traumatic or morbid experiences), as well as providing information on accessible mental health resources. Evidence-based digital and telehealth services that gained impetus during the COVID-19 pandemic were “strongly preferred by patients and families to emergency and inpatient care” [2]. Patient perceptions of telepsychiatry were overall positive and should be further assessed beyond the context of the current pandemic to account for changes in perception and impact on clinical outcomes [4]. Mental health care services are grappling with demand management strategies owing to an increased vulnerability to mental ill health [5].

The current pandemic has exacerbated the demand for mental health services, providing an opportunity for the supply of digital technologies (eg, videoconferencing-based tele-mental health interventions) and data-driven innovation (eg, predictive technologies), which are emerging to complement traditional in-person and telehealth aspects of psychology and psychiatry [6,7]. New digital technologies and data-driven innovation have been readily applied in radiology and dermatology (diagnostics and predictions with machine learning, robotics, and artificial intelligence [AI] platforms) [8]. However, there has been limited progress in digital mental health treatments, as evident from strong outcomes in efficacy trials since the early 2000s but ongoing failure in implementation owing to exceedingly reductive research models [9]. The Accelerated Creation-to-Sustainment model has addressed the research-to-practice gap; this model builds on existing methodologies, including human-computer interaction (HCI), implementation science, and trial methodology, and aims to develop and sustainably implement technology-enabled services [9].

Delays in progress persist owing to the low quantity and quality of information from evaluated evidence-based studies for digital mental health interventions. This is despite several reviews providing vast support to the potential of technology to improve their effectiveness, efficiency, cost, reach, personalization, and appeal [10]. Scholten and Granic [10] noted that researchers are reconceptualizing the scientific framework, methodology, and implementation strategies for digital mental health implementation and highlighted significant technological challenges including engagement, retention, fidelity, lack of personalization, and cognitive load. They proposed a design thinking solution to social scientists and clinical researchers. However, an impasse resulting from underlying disengagement issues is apparent, which has hindered the progress of evaluative research. Nebeker et al [11] provided a decision-making checklist to guide responsible digital technology selection in research, especially for dealing with technical and ethical considerations [11]. Nonetheless, clinical researchers subsequently called for further evaluation of this tool for its usefulness in technology selection. The pace of change in predictive technologies and other emerging aspects of digital mental health is intensified by the disparities and ineffectiveness of risk assessment in psychology and psychiatry (despite being reliable and valid).

It is difficult to capture the range of global innovation and research in digital mental health. However, Torous et al [12] provided an expert perspective on the depth of studies that contribute to the acceptability, feasibility, and early evaluation of digital mental health technologies. They acknowledged that telehealth is the appropriate solution for meeting the increased demand from the current pandemic, and they further emphasized an unprecedented potential for increased access and quality of digital mental health technologies [12]. Investment was noted as required with funding, research, policy changes, training, and equity, to enhance the progress of such innovations. Further research is required to validate the effectiveness and appropriateness of technology on a larger scale. The failed attempts of digital mental health researchers to engage interest, cooperation, and resolve from developers and their psychological and psychiatric counterparts to address the technical and ethical issues in evaluative research, signal the need to explore how to effectively integrate digital mental health solutions into mental health care service systems, and to apply these technologies among various populations.

## Risk Prediction and Predictive Technologies

Risk prediction in mental health has been underestimated as a methodology for counteracting mental ill health and suicidality owing to its confounding effects on clinical decision-making. With respect to the futility of risk assessment in psychiatry, Mulder et al [13] cautioned that suicide rates cannot be reduced through risk categorization from risk prediction in clinical practice by using risk assessment tools. They indicated the rarity of suicide and referred to the study by Chan et al [14], which identified the risk factors of high-risk groups, including individuals who have committed self-harm, and then evaluated risk assessment scales to assess the efficacy of these risk factors [14]. The lack of sufficient specificity and sensitivity of some the scales to be clinically useful was found to be counterproductive (eg, most of the low-risk categorized patients who completed suicide and high-risk groups of mainly false-positive findings) [14]. Few studies have prospectively examined the effect of risk assessment on patient outcomes [13]. Risk assessment tools may instill false reassurance in clinicians and managers; the unfeasibility of these tools needs to be highlighted, a qualitative understanding of the sequalae of suicidal ideation and attempts in an individual patient is required in a needs-based model of care, and risk assessments should be curtailed to minimize harm to the patient and the therapeutic alliance [13,14]. Redirection from statistical prediction of suicide (albeit valid and reliable in identifying risk factors for death by suicide) to a causal pathway has been recommended [13]. Instead, preventive interventions should focus on “real engagement with the individual patient, their specific problems and circumstances” [13]. This conundrum has been complicated by emerging predictive technologies, associated methodologies, and design systems.

AI approaches are being implemented in digital interventions and social suicide prevention initiatives (eg, web-based and smartphone apps), to enhance user experience and optimize personalized mental health care [15,16]. D’Alfonso [15] reported that data-driven AI methods could be employed to develop prediction and detection models for mental health conditions, gathering insight from digital exhausts (numerous personal digital device and social media interactions mined to obtain behavioral or mental health–related insights). Although limited by a lack of evaluated evidence-based clinical applications, AI has been portrayed as having remarkable accuracy in preliminary studies predicting suicide attempts [16]. Depression with psychosis, schizophrenia, suicidal ideation, and prior suicide attempts have been included as psychological risk factors. In a qualitative narrative review, D’Hotman and Loh [16] noted a significant potential to improve suicide prediction and prevention through novel analytical techniques and tools (eg, leveraging machine learning algorithms and data science) in coordination with the opportunity presented in contact and assessment by health services. They referred to a longitudinal study that reported that most people who die from suicide (83%) will have contacted health services in the year prior to their death, and 45% will have contacted these services in the month prior to their death, which highlights a challenge for more effective screening and tracking.

Although machine learning algorithms may improve existing decision support tools somehow, their usefulness in the clinical setting is limited by an ongoing lack of information on model building and uncertain accuracy [17-19]. Machine learning algorithms can potentially add value to the identification and diagnosis of mental health conditions (eg, depression, suicidal ideation, and cognitive decline), positive mental health outcomes (eg, resilience, identity formation, and personal growth) [17], and the prediction of problems [20]. However, these algorithms are not a replacement for explanations derived from data modeling; hence, hypothesis-based traditional research should not be dismissed. The scarcity of data of sufficient quantity and quality, often required to be able to leverage the potential of machine learning models, has contributed to developmental problems with this approach. There is no robust evidence on the transferability of machine learning models owing to a lack of institutional diversity in applying these models, annotation bottlenecks faced by supervised machine learning algorithms, sensitivity of health data, and privacy concerns [21]. A challenge for machine learning is the advancement of methodologies that integrate with other systems and the clinical validation of these methodologies [22]. Such methodologies are required owing to the rapid development of digital technologies that have outpaced the evaluation of rigorous digital health interventions [23].

## Extended Intelligence and Explainable AI

A fundamental problem is associated with the formulation of AI as a machine system distinct from humans, which attempts to control, design, and understand systems [24]. Ito [24] proposed extended intelligence as a design system that integrates humans and machines to constitute responsible, aware, and robust components of complex systems. A key issue is the capacity-to-sense in a manner that benefits humanity, leading to research on *veillance flux* for understanding, mediating, and augmenting the sensory capabilities of machines and humans and their connectedness to larger technological and societal systems [25]. The health care industry has lagged behind in the direct implementation of machine learning algorithms and complex models mostly because of issues with trust, generalizability, and transparency [26,27]. Explainable artificial intelligence (XAI) is an emerging methodology for endowing credibility, accountability, and trust in mission-critical areas of mental health [26]. The main advantage of XAI is that it combines common sense knowledge with semantic reasoning and causality models [26] for implementation in a manner that makes its functioning easy to understand [27]. Other benefits include transferability, informativeness, confidence, fairness, accessibility, interactivity, and privacy awareness [24]. Barredo Arrieta et al [27] proposed XAI as a core concept for responsible AI, which further encapsulates ethics, security, and safety concerns [27].

XAI facilitates a 3-way conversation among the patient, the health care practitioner, and the machine. XAI may help develop a mutually positive understanding and regulate, identify, and control the negative consequences of incorrect predictions or treatments. An issue with the use of existing decision support tools in physical health care (eg, AI-assisted clinical decision support systems) is that recommended treatments are often overridden by health care practitioners [28]. This is problematic if the outcome is ineffective and the decision cannot be adequately explained by the health care practitioner. It is important to consider and balance the differing interests and values in the ethical evaluation of AI-assisted decision-making [28]. The implications of different treatment recommendations may result in discordance (eg, clashing opinions of health care practitioner vs AI). XAI aims for a more complete understanding to potentially facilitate the decision-making process. In the mental health context, XAI is being applied in the conceptual stage of a knowledge-driven evidence-based recommendation system to improve surveillance for adverse childhood experiences as part of an early intervention approach [27]. The enhancement of the explanatory potential of health care practitioners’ decisions requires trials and evaluation with attention to privacy concerns [27]. XAI is worthy of further research, particularly in systems that generate predictions, for effective surveillance of functionality and implications in increasing human interaction with new technology.

## Other Emerging Aspects of Digital Mental Health

Accessibility, presentation, and transparency are important aspects of engagement with digital tools (eg, web-based and smartphone mental health apps) [5]. The ability of smartphones or smartwatches to capture the behavior and psychological symptoms of individuals with schizophrenia in real time and to elucidate their utility in early interventions of relapses may serve as a quantifiable method of calculating and managing risk [29]. Henson et al [29] reported that the integration of sensor data and relapse-detecting algorithms has a high potential for prediction and risk modeling. Resources for identifying safe and effective smartphone apps may help understand basic smartphone tools. A challenge for mental health care practitioners is to become familiar with these smartphone tools to fulfil the “next step in incorporating technology use into standard clinical care for relapse prediction and prevention” [29]. The major barriers to the integration of digital tools with other systems and clinical validation include an undeveloped risk-utility data governance framework [30] and an outdated and poorly developed information technology infrastructure (including data gathering and sharing) of many publicly funded health services [31].

The Internet of Things (IoT) refers to human-computer interconnectedness via smart objects or wearable devices, intermediary communication, and processing devices as well as hardware, fog, cloud, server infrastructure, or converged networks. The IoT has benefits in terms of monitoring, welfare interventions, and providing alerts and information services [32]. Digital phenotyping with the assessment of data from smartphones, consumer wearables, and social media has been scoped [33] but is yet to provide evaluated evidence regarding the detection and predictive capabilities of mental health interventions. Should the advantages of digital phenotyping be manifested in the clinical setting (eg, observing presented phenomena, opportunities for self-monitoring, and relapse prevention as well as potential interventions), then so will its disadvantages (eg, privacy in the management of personal data, and potentially not improving causal explanations or psychological understanding) [34].

Immersive virtual therapeutic interventions with virtual reality (VR), have been proposed as a cost-effective aid in treating mental health disorders and symptoms (eg, acrophobia [35], persecutory delusions [36], delusions, hallucinations, or cognitive and social skills associated with the schizophrenia spectrum [37]). A systematic review [37] referred to other studies on the high effectiveness and versatility of VR in the treatment of various pathological conditions (posttraumatic stress disorder, anxiety disorder, and specific phobias) [37]. However, available data on immersive VR are currently limited owing to the paucity of research on this topic. Further studies are required to ascertain transferability to this approach for other mental health conditions. Both VR and augmented reality systems have been recognized as very promising for future research [38], but their current scalability and accessibility inhibit their use in the COVID-19 pandemic [12].

Certain web-based alternatives including guided self-help, chatbots, and other web-based interventions can be used to increase affordable access to psychological therapies (either as an accompaniment or as stand-alone interventions). There is a lack of larger studies and clinical evaluation for evidence-based guided self-help interventions, although positive findings in reducing psychological symptoms in children [39] were expanded upon in the context of neurological illness [40]. The rarity and dissimilarity of evidence-based studies with chatbots limits discussions on their effectiveness. There is a need to develop culture-suitable chatbots and to evaluate software that simulates a conversation via AI with a human on text messaging or voice chat platforms (natural language processing) [41]. A scoping review referred to early evaluation of the feasibility and acceptance of chatbots, and its randomized controlled design yielded effective chatbots with benefits especially directed toward well-being, stress, and depression [42]. However, Bendig et al [42] raised questions regarding the security and acceptance of such new technologies, with issues based on commercial conflict of interests, objective comprehensibility, nonclinical pilots, conceptualization for complex psychotherapeutic understanding (eg, suicidal ideation), and replicability [42]. A range of other web-based interventions is available; for example, TogetherAll, a Canadian anonymous digital mental health service, and Head to Health, an Australian digital mental health gateway providing trusted, evidence-based, peer-reviewed nationwide web-based resources.

No studies have assessed the effectiveness of mental health care facilities with an up-to-date and functional information technology infrastructure and the use of digital tools, digital phenotyping, IoT, and immersive technologies by capable digital mental health practitioners. A current state assessment of the Australian digital mental health ecosystem identified the integration of digital mental health services with the broader health system, software, platforms, and data and evaluation as critical supply issues [43]. The themes of digital inclusion and adoption of services, considerations for vulnerable cohorts, and utilization of lived experience in service design and delivery were identified as significant demand issues [43]. The objectives to improve service access (including workforce considerations) and reduce duplication of effort and investment involved sector consultation to inform the development of a framework [43]. However, a disclaimer for not making generalized representations of the appropriateness of the report [43] highlights transferability issues (for other ecosystems). The quagmire of determining effectiveness, validity, reliability, and reproducibility for digital mental health makes it necessary for engaged consultants to deflect responsibility, duty of care, or liability for other audiences relying on the information. The sense of excitement for integrating digital mental health with the delivery of mental health services to meet the increased demand is countered by the need for a more compelling evidence base for successful implementation and integration. Furthermore, there is no known register for digital mental health products or services to demonstrate the high quality, safety, security, transparency, and effectiveness of these products.

## HCI in Digital Mental Health

The emerging field of HCI is important for the efficacy of a hybrid model of care, especially for psychological screening and tracking with real-time automation and machine learning [5]. There is increasing focus on the digital therapeutic alliance (DTA), which extends from web-based and smartphone apps to digital mental health interventions [44]. Tremain et al [44] explored the DTA with researchers focusing on HCI and introduced psychologists to aspects of HCI. These authors proposed HCI to be conducive in expanding digital mental health, especially with smartphone apps. It remains a challenge to gain a better understanding of the nuances, particularities, and complexities of the psychology underlying the interaction between humans and AI. Furthermore, Tremain et al [44] suggested measuring smartphone interfaces and interactions to determine if there is a relationship between DTA and increased engagement with the adherence to digital interventions. In particular, the DTA was proposed to be measured by a hybrid or renewed version of the mobile Agnew relationship measure, thus accounting for HCI theories to inform affective computing, including the design for personalized responses and an appealing balance of the human characteristics of AI, such as traits, emotions, and intentions. It remains unknown how HCI would impact the efficacy of mental health care because of the limited studies on the DTA. D’Alfonso [15] suggested adapting from purpose-built measures focused on a human client and a computerized therapeutic intervention approach (eg, smartphone or web-based app or sophisticated conversational agent) fostered by AI [15].

## Digital Mental Health Implementation in a Hybrid Model of Care

The realization of the availability of digital mental health as a complementary approach is simultaneous with the transition into, and navigation through, the complex digital world. In 2017, a review of emerging digital mental health investigations (especially psychiatric rehabilitation) included web-based tools and smartphone apps and interventions, smartphone audio data analytics, digital self-management strategies, and statistical modeling [45]. Tal and Torous [45] reported that “new knowledge and evidence-based tools to better promote mental health diagnosis, treatment, rehabilitation, and recovery.” In aiming for progressive uptake and clinical adoption of digital mental health tools, Torous and Haim [46] detailed the advantages and disadvantages of dichotomous positions on digital solutions to transition a path towards embracing the opposing perspective. Gratzer et al [47] noted the potentially lasting benefits for a hybrid model of care in a review of digital mental health care delivery during the COVID-19 pandemic (eg, access, convenience, and adherence of telepsychiatry, internet-delivered cognitive behavior therapy, and apps) [47]. Potential drawbacks were noted, especially the lack of evidence regarding telepsychiatry for individuals with severe mental health disorders, high dropout rates with internet-delivered cognitive behavioral therapy with nonhuman therapists, and remarkably high dropout rates with apps [47]. It was further noted that standards for patient privacy, confidentiality, equity, and reliability of service delivery will need to be addressed through a thoughtful approach to sustain core services over the long term [47]. The delivery of more secure and stable platforms linked to AI-based apps for real-time screening, tracking, and treatment has been recommended to yield the best investment outcomes [47].

The integration of digital mental health solutions into pathways for delivering mental health services is gaining momentum particularly during the COVID-19 pandemic. For example, a primary youth mental health service has potential to boost care efficiencies in service entry, comprehensive assessment, multidisciplinary care, and routine outcome-based monitoring [48]. Davenport et al [48] referred to extensive co-design and clinical research leading up to the concept of digitally flipping clinics, resulting in a screening and tracking flow chart and operation protocol that includes plans for addressing care needs, categorization in accordance with symptom intensity, and instructions for further assessment and review. A digital platform serves as a customizable digital toolkit in a fee-for-service early intervention system [48]. Previous mental health screening programs in low-prevalence settings have yielded controversial findings owing to a high rate of false-positive results, with a suggestion for screening being more practical in high-prevalence settings [49]. However, an evaluation of the effectiveness of screening in a prison setting was considered as being required in terms of its potential advantages and disadvantages [49]. An evaluation of screening and treatment for depression at the workplace found these approaches to be cost-effective [50]. However, more economic evaluations are required owing to the low rates of treatment uptake, or the lack of effective available interventions could impair the cost-effectiveness of mental health screening.

The inequities in mental health care (eg, the inconvenience of distance and inferior internet connectivity) are a common issue [47,51]. In consideration of demand outstripping supply, the complexities of user experience from clinician and patient perspectives [51] including in-patient care and environmental considerations should be considered with importance (eg, disentangling addiction, psychosis, and serious mental illness from subsyndromal conditions, common mental disorders, or false cases). An advantage of digitalization is that a negative effect of incompatible in-patient care is replaced by unobtrusive solutions which are not as limited (eg, qualification and capabilities related to administration and assessment). The disadvantages of digitalization—in terms of social justice—are compromised accessibility and functionality for individuals with severe mental health disorders [51] and constraints in understanding gestural components (eg, behavioral observation).

Data from the general population and subpopulations are yet to be effectively integrated with algorithms that contribute to a hybrid model of care as part of a general guide [5]. Machine learning and deep learning have until recently only been fed structured data in medical informatics, neglecting invaluable information from unstructured clinical notes [52]. Unstructured data from medical records combined with deep learning tools hold promise in identifying the most suitable candidates for a study on youth depression, but a reliable recommendation system has not been established [53]. Machine learning has had limited advantages associated with structured data. A study predicting the outcomes of stroke [54] reported that this was because of study conduct and reporting; clinical prediction tools have not made their models available for use or evaluation [54]. The ability of AI to enhance risk prediction in mental health assessment may extend from mining unstructured data (eg, through text or continuous sensor monitoring), such as a study on sepsis, which revealed improved algorithm accuracy (deidentified data made available for review) [55]. The implementation of algorithm prediction systems in predicting the risk of suicide is currently limited to experimental and feasibility studies (with utility) without efficacy evaluation [16,56-58].

The challenges associated with the implementation of digital health solutions (including mental health) are mostly related to their integration with standard clinical care. Various solutions such as apps are available; however, methodologies have not evolved alongside them to perform timely, cost-effective, and robust evaluations [59]. Guo et al [59] recommended innovative approaches, such as simulation-based approaches, for end-users to holistically resolve this dilemma. A main finding is that developers need assurance of a reasonable reward (by means of a valid market) for their time and effort and to not be perturbed by the fallacy of traditional approaches [59]. Further emphasis should be laid on the transparency and reproducibility of machine learning–based prediction models [60]. Researchers can help in generating their code and preferably making their data available. Reporting standards may discourage digital health systems from being commercially available before complete evaluation. The agility of machine learning [17] renders it cumbersome and unfair for a long series of randomized controlled trials. For example, a quasi-experimental design for a model of delivering technology-enabled mental health services revealed relatively strong evidence on model effectiveness before evaluation through randomized controlled trials [61].

Further innovation is required to overcome challenges, increase efficiency, and generate new opportunities to compensate for general human deficiencies, thus helping served, underserved, and unserved individuals [51]. Jurisdictions changed regulatory and compensatory frameworks to allow providers and patients more flexibility in their care options during the COVID-19 pandemic [47]. However, policies regarding the expanded access to treatment are required (eg, protection of privacy and allowing for continued compensation for virtual care) [51]. A hybridized approach with HCI and XAI has the potential for a superior ability to rapidly adapt and effectively recognize, acknowledge, and address marginalized, distorted, acquiesced, and subsyndromal cases, among others. Training for mental health care practitioners in delivering digital mental health interventions [51] should focus on active listening and observation skills to identify prejudice and stigmatization, guilt, fear, shyness, discomfort, shame, detachment, embarrassment, social anxiety, and social isolation among other issues. A reliable and valid understanding of the fundamentals and points of failure is needed to drive innovation. However, we do not see any panacea for dealing with disclosure or dissatisfaction around these issues.

## Digital Mental Health Insights and Ethical Considerations

The relatively slow progress in furthering the evaluation of digital mental health has hindered its progress. The connections between stakeholders (patients or end users and physicians or mental health care practitioners) exist within a complex environment. Worldwide changes associated with the COVID-19 pandemic, notably demand outstripping supply, have presented a dilemma for decision-making related to mental health care with regards to digital mental health research and highly efficacious clinical trials. The conflict-theory of decision-making refers to the motivation to reduce tension during essential decision-making with forecasted difficulties regardless of which feasible option is chosen [62]. At the core of the theory is the premise that evasion or suppression of the dilemma is not an effective long-term solution. Psychologists, psychiatrists, and others concerned with mental health care delivery (especially policymakers) could seize the opportunity of digital mental health or dismiss it as euphoria, hype, or a threat without considering the unfavorable consequences of inaction. It is necessary to confront the challenges of digital mental health and determine the weightage of the advantages and disadvantages associated with its implementation.

The call for psychological self-assessments and brief interventions during the COVID-19 pandemic [3] is based on a patient perspective. However, the opposite viewpoint should also be considered. Despite the health-protective attributes of a physician’s career, the rates of depression, anxiety, and suicidal ideation are high among them (24.8% of a cohort of Australian physicians prior to a 12-month period—approximately 2-fold that of the general population) [63]. Gerada [63] described the “spiraling of discontent” and increased risk of suicide among physicians, which is associated with the development of psychological defenses that include depersonalization and dissociation [63]. Risk factors such as genetic predisposition and workplace stress contribute to an increased vulnerability to psychological distress and suicidal ideation among junior physicians [64]. The need for early intervention, a lack of discretely accessible and effective interventions, and the mobility of this group led to the development of the smartphone app Shift Health, which is focused on processing co-design and user feedback to engage users and ensure user adherence with increased effectiveness and appropriate screening, tracking, and treatment [64]. The advantages of this app are improved accessibility, cost-effectiveness, usefulness for larger cohort studies, and the ability to inform organizational solutions. The disadvantages of this app include overcoming of the barriers to app usage (eg, personal selection of modules and continuous cohort engagement), attrition rates, and engagement with clinical researchers for evaluation on a larger scale. A strategy for engaging clinical researchers in external evaluation is crucial for its effectiveness beyond strong efficacy in clinical trials.

A digital platform for the management of mental ill health requires innovative design and delivery to provide the capacity for engagement and shared decision-making among users [65]. Torous et al [66] supported the evolution of the therapeutic relationship among users with a freely shared platform code to encourage adaptation and improvement, especially in digital phenotyping. Connectivity to third-party apps using application programming interfaces provides the opportunity to extend the ecosystem of the platform and its capabilities. A knowledge and learning platform should include blueprint skills, inductions, and learning sessions. Dissemination of technical skills with training, weblinks, and support would help others master these skills. Digital innovation requires development along with simultaneous learning in the workflow, with suitable tools and guides available to encourage innovation. It is important for there to be easy access to reports, virtual classrooms, and demonstrations of content and video creation, use of a dashboard, and the creation of course offerings. It is recommended to seek assistance or self-help for creating and setting up of digital content. Furthermore, blogs, podcasts, webinars, and in-person or digital sessions are required to share effective, valid, and reliable data and knowledge.

It is important for strategies to enhance resilience particularly considering the stress of the COVID-19 pandemic [67]. It is also important to address ethical challenges in mental health and suicide prediction tools and models [16,51] with particular attention on privacy, confidentiality, fairness, transparency, and accountability [68]. Private industries should be closely monitored by the scientific community through independent reviews and ethical oversight to confirm the safety, effectiveness, and permissibility of their services [16]. Inputs from patients, service end-users, physicians, and caregivers should be factored in for developing solutions [15] as a first step to consider the consequences of machine learning and AI, including the need to reduce bias and increase efficacy. Different approaches are emerging, including a virtue ethics framework (ie, moral attention and appropriate extension of moral concerns) and micro-ethics (to understand and grasp the grittiness and distinctiveness of each situation) [69]. Impactful actions for using ethics and data security checklists are required with digital capabilities increasing at a rapid rate; for example, IoT-based approaches. Nabi [70] referred to bioethical agility and the need for a “shared conceptual mental map” to address the adverse issues in machine learning applications and to promote “beneficence, justice, patient autonomy, and to prevent maleficence” during the COVID-19 pandemic.

## Conclusion

Digital mental health is a debatable issue because of the uncertainty associated with it. The main advantage of this approach is its provision of a complementary system to address the urgent supply for increased demand. Furthermore, mental health care practitioners may be able to prevent mental ill health in some high-prevalence situations through early intervention with predictive systems. Digital platforms and AI-driven apps are currently the most important solutions in this regard. The disadvantages include a lack of evaluated evidenced-based studies. There are difficulties associated with digital mental health services to locate their niche especially during dominant reductivism in mental health research. However, these approaches are limited by a small pool of studies on their reliability, validity, and reproducibility. More generally, scientists have intended to separate machines from humans via AI and replace human decision-making with AI-based decision-making. On such a contentious topic, with continuous advancements in the field, the arguments for and against this topic will undoubtedly persist in the foreseeable future. However, in health care, the patient–health care practitioner relationship is of paramount importance, requiring a level of trust and understanding. XAI as a principal concept is worthy of investigation to help provide future direction. Furthermore, the potential financial implications of digital solutions are astronomical, such that current investment may eventually lead to evaluated scientific breakthroughs that will be largely accessible and affordable to the public.

Digital mental health with real-time screening, tracking, and treatment tools, especially platforms and web-based and smartphone apps, have yet to mature and are therefore not yet cost-effective. Nonetheless, promising developments have been reported, especially for youth mental health services. Digitalization and data science literacy and innovation may extend from resourceful web-based learning journeys. Human-centered outcomes through digital solutions in HCI require extensive consultation, detailed study (eg, DTA), and collaboration to develop and refine methodologies. The COVID-19 pandemic requires the mapping of issues and changes in categorization by systematically examining the uncertainty and impact of ideas, trends, and concepts, such that strategies can be developed in accordance with the relative effort, risk, and reward. The interface between data science and machine learning methods (pattern-based) and traditional research methods (hypothesis-driven) should be based on transparency and inclusiveness, policies, technologies, and education.

Digitalization and data science should instill a culture of quality, security, adaptability, technical excellence, positive empowerment, and motivation. End-users should instigate and simulate innovative research methods to overcome the difficulties of evaluation through traditional research methods. Modeling and prediction, along with future tools and processes, should borrow from existing ones to build new ones for future readiness (explore, interpret, and anticipate). The transparency and reproducibility of machine learning–based prediction models has been emphasized. The technical and ethical challenges, as well as the risk of digital and data-oriented tools, require a balance with opportunities and strategic research and investment. Innovative, safe, and secure digital mental health implementation is needed to improve public health (including equity) and respond to the demand outstripping supply of mental health resources. It is important to engage with eminent digital mental health in a hybrid model of care owing to its positive impact on resilience. Convergence on methodologies is required for expedited evaluation of rigorous preventive strategies and interventions.

## Abbreviations

AI: artificial intelligence
DTA: digital therapeutic alliance
HCI: human-computer interaction
IoT: Internet of Things
VR: virtual reality
XAI: explainable artificial intelligence
